# MicroRNA-222 promotes drug resistance to doxorubicin in breast cancer via regulation of miR-222/bim pathway

**DOI:** 10.1042/BSR20190650

**Published:** 2019-07-16

**Authors:** Hong Dai, Ling-yun Xu, Qi Qian, Qiu-wei Zhu, Wei-xian Chen

**Affiliations:** Department of Breast Surgery, the Affiliated Changzhou No. 2 People’s Hospital of Nanjing Medical University, Changzhou 213000, China

**Keywords:** Breast cancer, Chemoresistance, Doxorubicin, miR-222

## Abstract

Resistance to doxorubicin (DOX) is the most common clinical problem in breast cancer therapy, and the underlying molecular mechanism remains to be investigated. MicroRNAs (miRNAs) exhibit important regulatory functions in various malignant tumors including breast cancer. The aim of the present study was to find the relationship between *miR-222* and DOX resistance. We found that *miR-222* was highly expressed in patients’ serum and DOX-resistant cell line MCF-7-R and that *miR-222* could promote proliferation and migration of breast cancer cells. Our results also showed that inhibition of *miR-222* in MCF-7-R significantly increased Bcl-2 interacting mediator (Bim) expression both in mRNA and protein levels by using quantitative real-time PCR (qRT-PCR) and Western blot. MTT and flow cytometry suggested that lower expressed *miR-222* enhanced apoptosis and decreased IC_50_ of MCF-7-R cells. Conversely, in MCF-7 cells transfected with *miR-222* mimics, up-regulation of *miR-222* was associated with decreased Bim level accompanied by less apoptosis and higher IC_50_. Moreover, *miR-222* inhibitors reversed DOX resistance via *miR-222*-Bim-caspase pathway. Collectively, these data first elucidated that *miR-222* could function as an oncogene and was able to reduce the sensitivity of breast cancer cells to DOX through *miR-222*-Bim-caspase pathway, which provided a potential target to increase DOX sensitivity in clinical breast cancer treatment.

## Introduction

Breast cancer is considered as the most common malignant tumor among women worldwide [1]. Chemotherapy is a crucial option for those patients in late stage. Unfortunately, patients rapidly get drug resistance to chemotherapy, which leads to a high incidence of early relapse [2]. Doxorubicin (DOX) is an extensively used anti-tumor agent in multifarious cancer treatment including breast cancer. However, the cardiotoxicity, low-selectivity, and drug resistance of DOX limited its extended application [3,4]. Therefore, reversing DOX chemoresistance needs more efforts to make.

MicroRNAs (miRNAs) are small non-coding RNAs (typically 19–22 nucleotides) that function generally in regulating their target gene expression at the post-transcriptional level [5]. MiRNAs could function as both oncogenes or tumor suppressors on approach to regulating genes in different cells [6]. Moreover, selective expression of miRNAs in many cancer diseases promoted therapeutic drug resistance in conventional chemotherapy. Therefore, miRNAs are significant for cancer progression, which may serve as alternative targets in treating breast cancer as well as reversing drug resistance [7]. In our previous work, *miR-222* has already been reviewed as a promising biomarker for breast cancer development and progression [8]. Through miRNA microarray and experiments in MCF-7 breast cancer cell line and DOX-resistant variant, we have found that *miR-222* was responsible for DOX-resistance [9]. However, the underlying molecular mechanisms remain unclear.

In the present study, *miR-222* was demonstrated to participate in the growth and migration of breast cancer cells. Up-regulated expression of *miR-222* was also found in breast cancer patients. Furthermore, it was evident that *miR-222* predicted sensitivity to DOX, considering its up-regulation leading to DOX resistance.

## Materials and methods

### Sample sources and cell lines

Blood samples from 25 healthy subjects and 25 breast cancer patients from the Affiliated Changzhou No. 2 People’s Hospital of Nanjing Medical University were collected and approved by the human protection subjects committee for the present study. MCF-7 breast cancer cell line and DOX-resistant variant (MCF-7-R) were purchased from the Shanghai Gaining Biotechnology Co., Ltd. Thereinto, MCF-7-R was established by adding high concentrations of DOX (Sigma–Aldrich) into MCF-7 cell culture medium, according to the manufacturer’s instructions. MCF-7 cells were cultured in RPMI 1640 medium (Gibco BRL) containing 10% heat-inactivated fetal bovine serum (FBS) and 1% penicillin–streptomycin antibiotic mixture, and MCF-7-R cells were maintained in DMEM/F12 medium (Gibco BRL) containing 10% heat-inactivated FBS and 1% penicillin–streptomycin antibiotic mixture. Culture conditions were both 37°C, with 5% carbon dioxide and 95% air.

### RNA samples and transfection

Human *miR-222* mimics, *miR-222* inhibitors, negative control oligonucleotides (NCO), and Bcl-2 interacting mediator (Bim) siRNA were obtained from the BOHAO Co., Ltd (China). The sequences of RNAs: *miR-222* mimics, 5′-AGCUACAUCUGGCUACUGGGU-3′; *miR-222* inhibitors, 5′-ACCCAGUAGCCAGAUGUAGCU-3′; NCO, 5′-AUCCCAUGGUGGGUUACAUGGUU-3′; and Bim siRNA, 5′-GACCGAGAAGGUAGACAAUUU-3′. Lipofectamine 2000 (Invitrogen, U.S.A.) was used to transfect RNAs into cells at a final concentration of 50 nM.

### Quantitative real-time PCR

The expression levels of *miR-222* and Bim were determined by quantitative real-time PCR (qRT-PCR). TaqMan MicroRNA Detection Kit was purchased from the Thermo Fisher company. Among them, U6 snRNA was used as an internal reference indicator for *miR-222* expression in qRT-PCR quantitation, while GAPDH was used as an internal reference index of Bim expression in qRT-PCR quantitation. At last, 2^−ΔΔ*C*^_t_ method was applied to calculate *miR-222* and Bim levels.

### Luciferase reporter assay

In order to generate the wild-types, Bim 3′-UTR was cloned into the pMIR-REPORT™ miRNA Expression Reporter Vectors. The mutant-type was created by mutating the seed regions of the *miR-222*-binding sites (UGAAUGU to UGAUAGU) with the Site-Directed Mutagenesis Kit (Takara, Japan). Double-luciferase reporter assay (Promega, Madison, WI, U.S.A.) was used to study the interaction between *miR-222* and Bim. MCF-7-R cells were seeded in 12-well plates with the density of 3 × 10^5^, and 2 ml DMEM containing 10% FBS but without any antibiotics were added. When cells reached 70–80% fusion degree, *miR-222* and Bim 3′UTR or Bim 3′UTR mutants would get a co-transfection. Subsequently, luciferase assay was performed according to the following method: (i) cell culture fluid was discarded, 100 microns report lysis buffer was added to each hole, and the chamber was shook for 15 min. (ii) The cells were collected into a 1.5-ml centrifuge tube and repeatedly freeze-thawed in liquid nitrogen for two times; (iii) balanced at 22°C with a water bath for 10 min; (iv) centrifuged at the conditions of 12000×***g***, 4°C for 2 min; and (v) added 20 μl cell lysate and 100 μl luciferase reaction substrate, mixing it well, and determining the values.

### Western blotting analysis

MCF-7 cells in the logarithmic phase were collected and the cellular proteins were extracted. After that, 10% separation adhesive and 4% concentration adhesive were prepared, and SDS/polyacrylamide gel was placed in an electrophoresis tank for sample loading, electrophoresis and protein transfer. Subsequently, polyvinylidene difluoride (PVDF) membranes were cleaned with Tris-buffered saline (TBS)-Tween (TBST) for three times, each time for 10 min. After blocking with 5% skim milk powder in TBST for 2 h, the membranes were incubated with primary antibodies at 4°C overnight followed by secondary antibodies. The antibodies against Bim, caspase-3, caspase-9, and β-actin were obtained from Cell Signaling Technology.

### Cell viability and proliferation

MCF-7 cells were transfected with *miR-222* mimics or inhibitors at a final concentration of 50 μM. After 48 h of transfection, fresh medium containing different concentrations of DOX was used to replace the medium. Cell viability was measured. MTT assay was performed to calculate 50% cell death (IC_50_) of DOX. The ^3^H thymidine incorporation assay was used to determine cell proliferation during the last 6 h of incubation.

### Cell migration *in vitro*

MCF-7 cells were transfected with *miR-222* mimics or inhibitors for 48 h. Before seeding, the under-surface of Transwell’s upper chamber was coated overnight with collagen I at 4°C, and then 1 × 10^5^ cells were inoculated in serum-free medium of Transwell’s upper chamber. Then, porous transparent polyethylene terephthalate membrane with 8-micron pore diameter (Corning Costar Corporation, U.S.A.) and hydroxyurea (Sigma–Aldrich) was added to prevent cell proliferation. Approximately 24 h later, the cells on the under-surface of upper unit were fixed, stained, and counted under a phase contrast microscope.

### Bioinformatics and statistical analysis

The online miRNA database TargetScan (http://www.targetscan.org) was used for prediction of *miR-222* target gene. All experiments were carried out at least three times. One-way analysis of variance (ANOVA) followed by the Student–Newman–-Keuls post-hoc test was used to assess the statistical significance of difference between cell groups. The comparison of miRNA expression level between serums from healthy subjects and breast cancer patients were performed using Wilcoxon’s unmatched pairs signed rank test. All the data were expressed as means ± standard deviation (SD) and analyzed for statistical significance using SPSS version 16.0. *P*<0.05 was considered statistically significant.

## Results

### *miR-222* is highly expressed in MCF-7-R cells and patients’ serums

**Figure 1:**
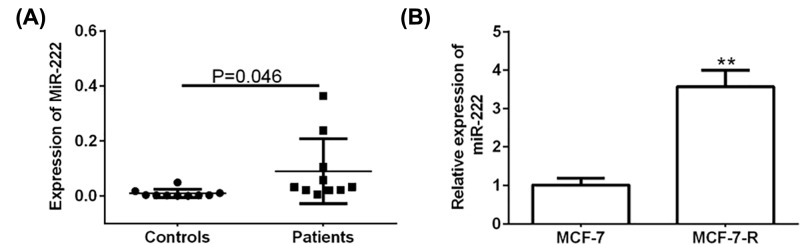
*miR-222* is highly expressed in MCF-7-R cells and patients’ serums (**A**) The expression of *miR-222* in serums of breast cancer patients and healthy controls. (**B**) The expression of *miR-222* in MCF-7 and MCF-7-R breast cancer cell lines. **P<0.05.

Expressions of *miR-222* were analyzed by RT-qPCR in serums from patients and healthy controls. Compared with normal controls, *miR-222* was obviously up-regulated in breast cancer patients’ serums (Figure 1A). Besides, data showed a relative higher expression of *miR-222* in MCF-7-R breast cancer cells with respect to MCF-7 cells (Figure 1B). Therefore, *miR-222* was suggested as a tumor promoter in breast cancer cells and may be associated with DOX resistance in MCF-7.

### *miR-222* promotes proliferation and migration of breast cancer cells

**Figure 2:**
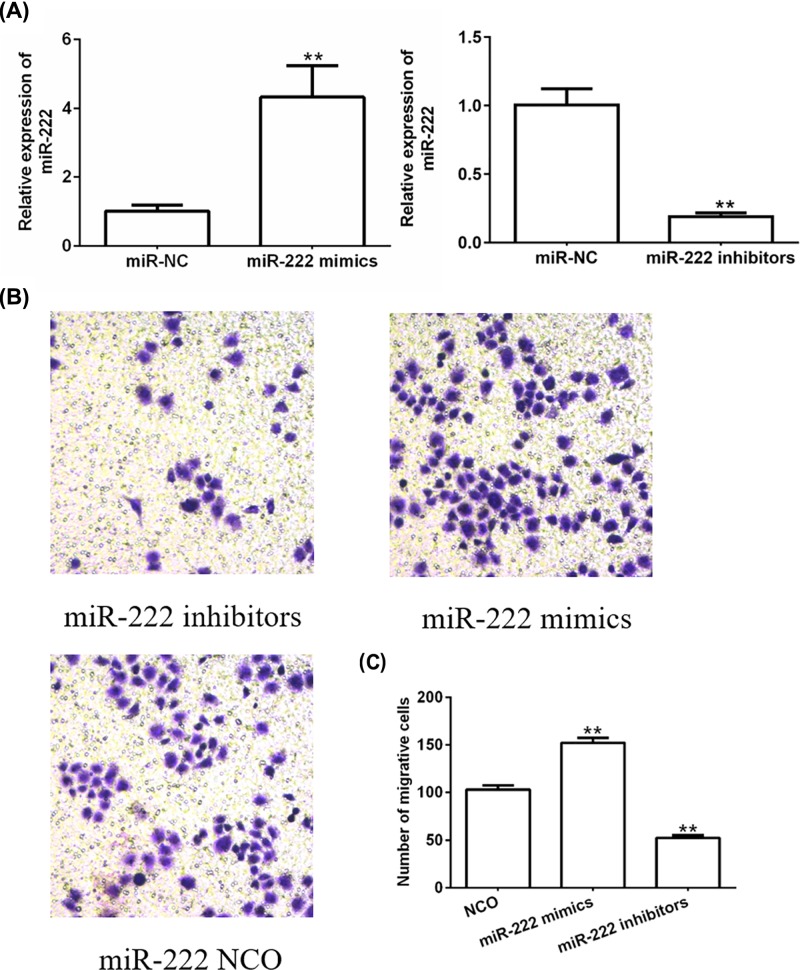
*miR-222* promotes proliferation and migration of MCF-7 cells (**A**) The level of *miR-222* was analyzed by PCR after MCF-7 cells were transfected with *miR-222* mimics, inhibitors, and negative controls for 48 h. **P<0.05 vs. control group. (**B**) Cell proliferation was measured using ^3^H thymidine incorporation assay after MCF-7 cells were transfected with *miR-222* mimics, inhibitors, and negative controls for 48 h. (**C**) Migration was analyzed using Transwell assays after MCF-7 cells were transfected with *miR-222* mimics, inhibitors, and negative controls for 48 h. **P<0.05 vs. control group.

In order to figure out the function of *miR-222* in breast cancer progression, we transfected *miR-222* mimics or inhibitors to change the level of *miR-222* in MCF-7 cells. It was found that *miR-222* level was increased sharply to ∼10.22-fold after introducing *miR-222* mimics, and *miR-222* level was decreased to ∼4.27-fold after introducing *miR-222* inhibitors (Figure 2A). Moreover, MCF-7 cells showed clearly enhanced proliferative capacity in *miR-222*-overexpressing group and significantly weakened proliferative capacity in *miR-222*-knockdown cells (Figure 2B). Furthermore, the migration of MCF-7 cells was significantly promoted when overexpressing *miR-222*, and was obviously inhibited when knocking down *miR-222* (Figure 2C). These results indicated that *miR-222* could promote cell proliferation and migration in breast cancer cells.

### *miR-222* is connected with DOX resistance of breast cancer cells

**Figure 3:**
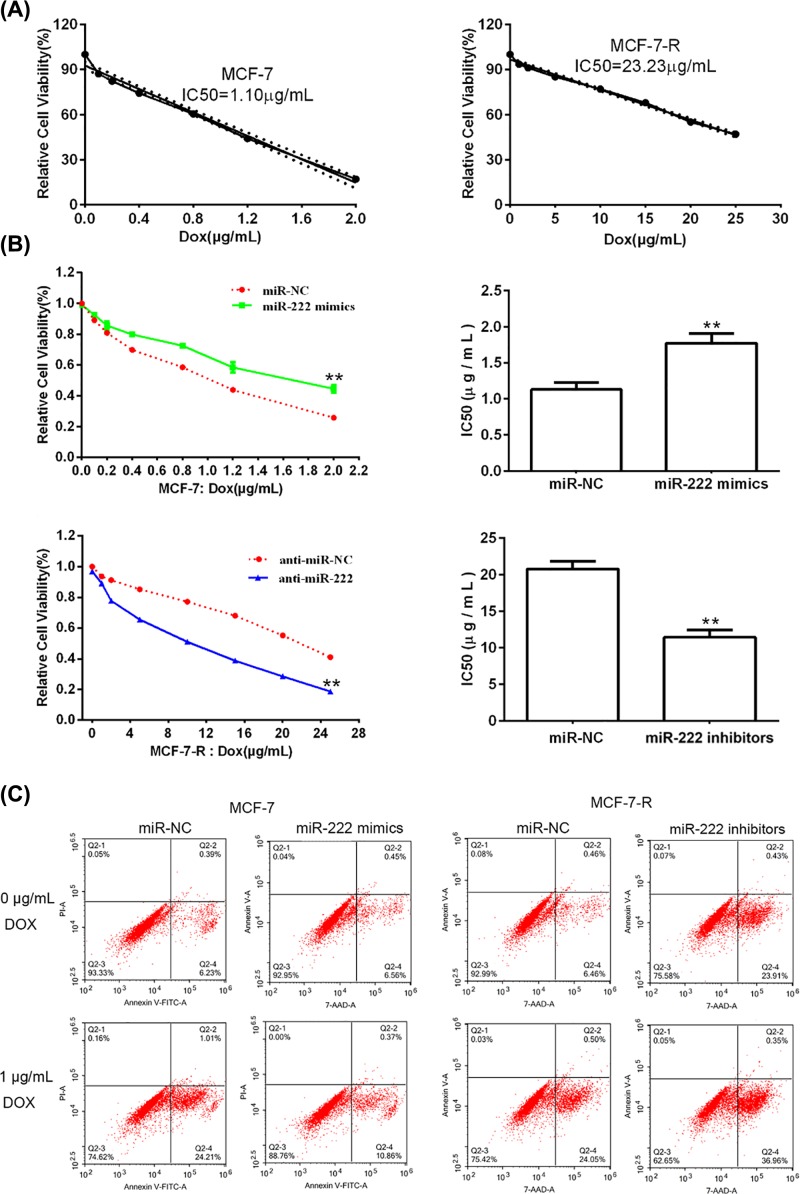
*miR-222* is connected with DOX resistance of breast cancer cells (**A**) MTT was used to calculate the IC_50_ of DOX in MCF-7 and MCF-7-R cells. (**B**) MTT was used to measure the IC_50_ of DOX after MCF-7 cells were transfected with *miR-222* mimics or negative controls, and after MCF-7-R cells were transfected with *miR-222* inhibitors or negative controls for 48 h. **P<0.05 vs. control group. (**C**) Flow cytometry was used to evaluate the apoptotic rates after MCF-7 cells were transfected with *miR-222* mimics or negative controls, and after MCF-7-R cells were transfected with *miR-222* inhibitors or negative controls for 48 h.

To confirm the relationship between *miR-222* and DOX sensitivity of breast cancer cells, we used a DOX-resistant cell line model (MCF-7-R) through the way exposing MCF-7 cells to high concentration of DOX (Figure 3A). It was observed that IC_50_ of DOX was obviously increased in MCF-7 cells transfected with *miR-222* mimics and significantly decreased in MCF-7-R cells transfected with *miR-222* inhibitors (Figure 3B). Low expression of *miR-222* made MCF-7-R sensitive to DOX while high expression of *miR-222* made MCF-7 resistant to DOX. We further examined whether DOX sensibility regulated by *miR-222* was connected to cell apoptosis. As shown in flow cytometry (Figure 3C), MCF-7-R cells reduced survival and increased apoptosis when treated with *miR-222* inhibitors, with respect to anti-miR-NC group. When treated with DOX, MCF-7-R cells with *miR-222* inhibitors showed a decreased survival and elevated apoptosis in respect to anti-miR-NC. Therefore, reducing *miR-222* level made more MCF-7-R cells apoptosis induced by DOX.

### *miR-222* regulates the level of Bim in MCF-7 cells

**Figure 4:**
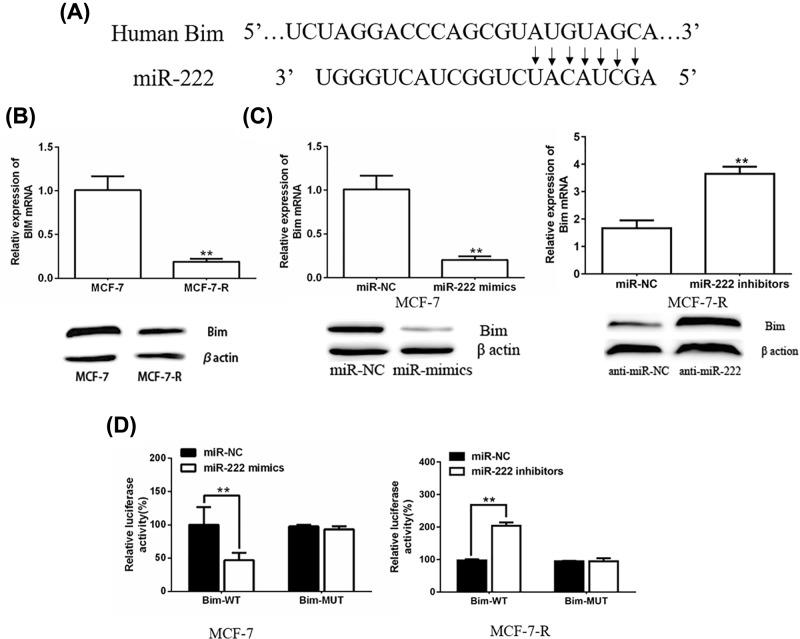
*miR-222* regulates the level of Bim (**A**) *miR-222* targets the Bim gene. (**B**) The expression of Bim was evaluated by PCR and Western blot in MCF-7 and MCF-7-R cells. **P<0.05 vs. MCF-7 group. (**C**) The expression of Bim was evaluated by RT-qPCR and Western blot after MCF-7 cells were transfected with *miR-222* mimics or negative controls, and after MCF-7-R cells were transfected with *miR-222* inhibitors or negative controls. **P<0.05 vs. control group. (**D**) Luciferase reporter assays was performed after MCF-7 cells were transfected with wild-type or mutant Bim 3′-UTR-reporter constructed together with *miR-222* mimics or negative controls, and after MCF-7-R cells were transfected with wild-type or mutant Bim 3′-UTR-reporter constructed together with *miR-222* inhibitors or negative controls. **P<0.05 vs. control group.

To explain why *miR-222* is connected to DOX sensitivity of MCF-7, we searched TargetScan database (http://www.targetscan.org/) and discovered that *miR-222* may target the Bim gene. The position 4052-4059 of Bim 3′-UTR was complementary (5′…AUGUAGC…3′) to the sequence of *miR-222* (Figure 4A). Western blot showed that the level of Bim was obviously lower in MCF-7-R cells than in parental MCF-7 cells (Figure 4B). Therefore, reduced Bim expression might be a potential reason for DOX resistance of MCF-7-R cells. We transfected *miR-222* mimics to MCF-7 cells and *miR-222* inhibitors to MCF-7-R cells in order to change *miR-222* level. As we expected, both Bim mRNA and protein levels were decreased in MCF-7 cells transfected with *miR-222*-mimics. Meanwhile, Bim mRNA and protein levels were both pronounced increased in MCF-7-R cells transfected with *miR-222* inhibitors (Figure 4C). A luciferase reporter vector was then constructed to investigate whether *miR-222* directly targeted Bim. The results showed that *miR-222* mimics group had a significantly decreased luciferase activity of wild-type in MCF-7 cells with respect to control group. Meanwhile, the relative luciferase activity of wild-type was significantly up-regulated in *miR-222* inhibitors group in MCF-7-R cells (Figure 4D). No phenomenon was observed in mutations of Bim. These data indicated that *miR-222* negatively regulated Bim expression.

### *miR-222* inhibitors reverse DOX resistance via *miR-222*-Bim-caspase pathway

**Figure 5:**
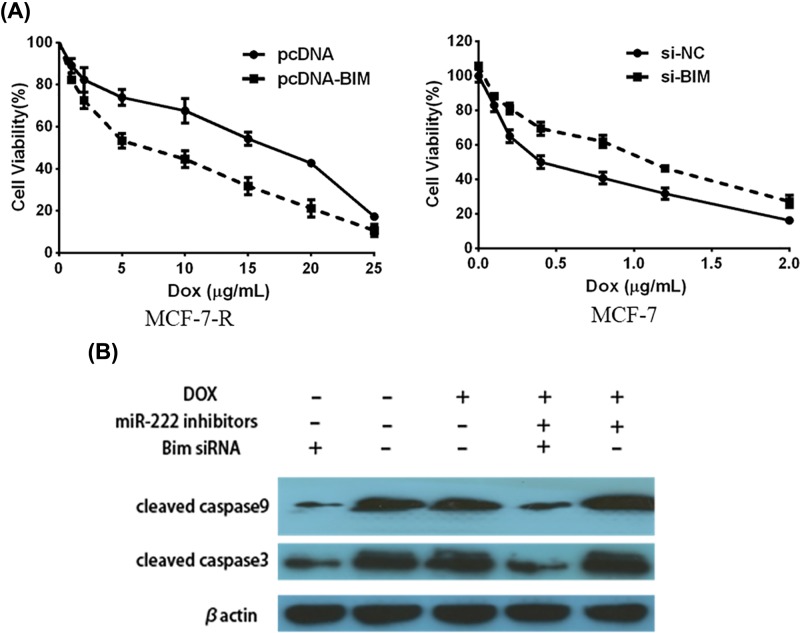
*miR-222* inhibitors reverse DOX resistance via *miR-222*-Bim-caspase pathway (**A**) Cell viability was measured by MTT assay after MCF-7-R cells were transfected with Bim mimics or negative controls, and after MCF-7 cells were transfected with Bim inhibitors or negative controls for 48 h. (**B**) Western blot analysis was performed to measure the activation of caspase-9 and caspase-3 after MCF-7-R cells were treated with Bim inhibitors, *miR-222* inhibitors, with or without the presence of DOX.

We overexpressed Bim in MCF-7-R cells and down-regulated Bim in MCF-7 cells. MTT assay showed that Bim overexpression reversed DOX resistance of MCF-7-R cells. MCF-7 cells had resistance to DOX while Bim inhibition (Figure 5A). We finally investigated the Bim regulation pathway by using *miR-222* inhibitors in MCF-7-R cells. Bim is an important member in pro-apoptotic Bcl-2 family proteins [10]. It can induce the release of cytochrome *c* from mitochondria and activate caspase to apoptotic protein in many cells. Western blot showed that down-regulated Bim or *miR-222* could activate caspase pathway (Figure 5B). In addition, Bim siRNA significantly inhibited the cell death induced by *miR-222* inhibitors with DOX in MCF-7-R cells. It was therefore demonstrated that Bim pathway was able to reverse DOX resistance in MCF-7 cells.

## Discussion

MiRNAs play an important role in cancer therapeutic response and spontaneous acquired resistance. Development of therapeutic approaches based on miRNAs has made considerable achievements. Here we reported that *miR-222* induced acquired resistance to DOX in breast cancer cells. Accumulating evidence suggests that *miR-222* may act as an oncogene in various cancer types. For instance, up-regulating *miR-222* promoted recurrence and reduced disease-specific survival in bladder cancer [11]. It was reported that high expression of *miR-222* induced proliferation, cell cycle distinction, and migration capacity and suppressed apoptosis of prostate cancer cells [12]. Besides, *miR-222* was proved to target ARID1A to enhance proliferation and invasion of cervical cancer cells [13]. Furthermore, *miR-222* was found to be responsible for resistance of tamoxifen which is widely used for breast cancer treatment [14]. However, the specific functions related to *miR-222* remains unknown in breast cancer.

In the present study, we have found that *miR-222* was highly expressed in breast cancer patients’ serums and especially in MCF-7-R cells. Moreover, these results were positively associated with cell proliferation and migration *in vitro*, indicating that *miR-222* may be a drug resistance promoter in MCF-7-R. Much efforts have been devoted to demonstrate that targeting diverse miRNAs for therapy may improve the anti-tumor effect of chemotherapeutic drugs. For example, the sensitivity to DOX was significantly elevated after transfecting anti-miR-21 oligonucleotide to glioblastoma cells [15]. Down-regulating miR-26b expression inhibited anti-apoptotic gene expression to enhance the curative potency of hepatocellular carcinoma cells to TRAIL [16]. miR-193b was proved to induce hepatoma cell apoptosis following cisplatin treatment. In the present study, it was revealed that knockdown of *miR-222* significantly made MCF-7-R cells re-sensitive to DOX.

Bim is called Bcl-2 interacting mediator of cell death, which is known as B-cell chronic lymphocytic leukemia-lymphoma-like 11 (BCL2L11) [17]. Bim acts as a key to regulate the mitochondrial (intrinsic) apoptotic pathway, directly activating the pro-apoptotic effect, and promoting cell apoptosis by binding to all of Bcl-2 family members [18]. Our results showed that transfecting *miR-222* inhibitors to up-regulate Bim in chemoresistant cancer cells reversed sensitivity of MCF-7-R cells to DOX. Our studies also confirmed that mitochondrial (intrinsic) apoptosis was under control of the Bim pathway. The up-regulation of Bim mediated by *miR-222* inhibitors in MCF-7-R cells significantly promoted DOX to damage mitochondria. Therefore, after caspase-3 was resolved, the biomarker of intrinsic apoptosis caspase-9 was activated.

## Conclusions

In summary, the present study lay the foundation that *miR-222* is a novel oncogene which promotes the chemoresistance in breast cancer. Our research also indicated that targeting the *miR-222*-Bim pathway may be a potential therapy for breast cancer in the future.

## Abbreviations

Bim: Bcl-2 interacting mediator
DOX: doxorubicin
FBS: fetal bovine serum
miRNA: microRNA
NCO: negative control oligonucleotide
qRT-PCR: quantitative real-time PCR
TBST: Tris-buffered saline (TBS)-Tween
